# Perceptions about the harm of secondhand smoke exposure among U.S. middle and high school students: findings from the 2012 National Youth Tobacco Survey

**DOI:** 10.1186/1617-9625-11-16

**Published:** 2013-07-17

**Authors:** Brian A King, Shanta R Dube, Stephen D Babb

**Affiliations:** 1 Office on Smoking and Health, National Center for Chronic Disease Prevention and Health Promotion, Centers for Disease Control and Prevention, 4770 Buford Highway, MS K-79, Atlanta, GA 30341, USA

**Keywords:** Tobacco, Tobacco smoke pollution, Adolescent, Perception, Risk, Students

## Abstract

**Background:**

Increased knowledge of the harmful effects of SHS is an evidence-based key indicator for eliminating nonsmokers’ exposure to SHS. This study assessed the prevalence and predictors of perceptions about the harm of secondhand smoke (SHS) exposure among U.S. middle and high school students.

**Findings:**

Data were obtained from the 2012 National Youth Tobacco Survey, a nationally representative school-based survey of U.S. students in grades 6–12. Respondents who reported that they thought breathing smoke from other people’s cigarettes or other tobacco products causes “some” or “a lot” of harm were considered to have the perception that SHS is harmful. Multivariate logistic regression was used to identify predictors of the perception that SHS is harmful. Predictors included sex, race/ethnicity, school grade level, current tobacco use, and whether the respondent currently lived with a tobacco user. Overall, 75.4% of students perceived SHS exposure as harmful. The adjusted odds of perceiving SHS exposure as harmful were higher among non-Hispanic Asians than among non-Hispanic whites, and among students in 10^th^-12^th^ grades than among students in 8^th^ grade. Adjusted odds were lower among boys than among girls, among non-Hispanic blacks than among non-Hispanic whites, among students living with a tobacco user than among those not, and among those who use combustible tobacco only or both combustible and non-combustible tobacco than among those who use no tobacco.

**Conclusions:**

Most middle and high school students perceive SHS exposure as harmful, but efforts are needed to increase the prevalence of this perception in certain subpopulations, particularly tobacco users.

## Findings

### Introduction

Exposure to secondhand smoke (SHS) from burning tobacco products causes disease and premature death among nonsmokers [1]. There is no risk-free level of SHS exposure, and even brief exposure can cause immediate harm [1]. During 2007–2008, approximately 88 million U.S. nonsmokers aged ≥3 years were exposed to SHS; among those aged 12–19 years, 46.5% (13 million) were exposed to SHS [2].

Increased knowledge of the harmful effects of SHS exposure is an evidence-based key indicator for eliminating nonsmokers’ exposure to SHS [3]. The continuum of change associated with eliminating nonsmokers’ exposure to SHS typically starts with increasing people’s knowledge of the hazards of exposure to SHS, changing their attitudes toward the acceptability of exposing nonsmokers to SHS, and enhancing their favorability toward smoke-free environments. Such changes can lead to increases in the adoption of, and compliance with, smoke-free environments as people become more conscious of the public health benefits of smoke-free air [3]. Parental attitudes and awareness toward SHS and its risks are associated with SHS exposure among youth [4], and greater perceptions of risk from SHS exposure among youth can reduce their likelihood of smoking initiation [5]. Although previous research suggests that a vast majority (95%) of U.S. adults recognize the hazards of youth exposure to SHS [6], no study has examined perceptions about the harm of SHS exposure among a representative sample of U.S. youth. Therefore, we assessed the prevalence and predictors of perceptions about the harm of SHS exposure among a nationally representative sample of U.S. middle and high school students who completed the 2012 National Youth Tobacco Survey (NYTS).

### Methods

#### Sample

NYTS is an ongoing school-based survey that collects information on key tobacco-related measures from U.S. middle school and high school students [7]. Respondents are asked to complete a self-administered pencil and paper questionnaire in a classroom setting. Parental permission is obtained, and participation is voluntary at the school and student level. Among students, participation is anonymous. In 2012, n=24,648 respondents completed NYTS, yielding a response rate of 73.6%. No ethics approval was sought for this analysis because only secondary data were used.

NYTS utilizes a 3-stage cluster sampling procedure to generate cross-sectional, nationally representative samples of U.S. middle and high school students. The sampling frame consists of all public school, Catholic school, and other private school students in grades 6 through 12 in the 50 U.S. states and District of Columbia.

#### Measures

Perceptions about SHS harm were assessed by the question, “Do you think that breathing smoke from other people’s cigarettes or other tobacco products causes ‘no harm’, ‘little harm’, ‘some harm’, or ‘a lot of harm’?” Respondents who answered ‘some harm’ or ‘a lot of harm’ were considered to perceive SHS as harmful.

Respondent characteristics included: sex, race/ethnicity, grade level, tobacco use, and whether the respondent lives with a tobacco user. For tobacco use, combustible tobacco use was defined as the use of ‘cigarettes’, ‘cigars/cigarillos/little cigars’, ‘pipes’, ‘roll-your-own cigarettes’, ‘bidis’, ‘clove cigarettes’, or ‘hookahs or water pipes’ on ≥1 day during the past 30 days. Non-combustible tobacco use was defined as the use of ‘chewing tobacco/snuff/dip’, ‘snus’, or ‘dissolvable tobacco products’ on ≥1 day during the past 30 days.

#### Data analysis

Data were weighted to adjust for the differential probability of selection and response and analyzed using SAS-callable SUDAAN, version 9.2 (SAS Institute Inc., Research Triangle Park, NC). Point estimates and 95% confidence intervals (CI) were calculated overall and by each respondent characteristic. Differences between point estimates were considered statistically significant if confidence intervals did not overlap. Additionally, a binary logistic regression model was fitted with perceptions about SHS harm as the dependent variable; independent variables included sex, race/ethnicity, grade, tobacco use, and tobacco user living in the home.

### Results

Among all respondents, 4.7% reported that SHS exposure causes “no harm”, 19.9% reported SHS exposure causes “little harm,” 38.7% reported SHS exposure causes “some harm”, and 36.7% reported SHS exposure causes “a lot of harm” (Table 1). The prevalence of perceiving that SHS exposure causes “no harm” was significantly higher among boys (6.1%) than girls (3.3%), among non-Hispanic blacks (6.0%), Hispanics (6.3%), and non-Hispanic American Indians/Alaska Natives (10.9%) than non-Hispanic whites (3.6%), among those who used combustible tobacco only (9.1%) or both combustible and non-combustible tobacco (19.6%) than those who used no tobacco products (3.3%), and among those who lived with a tobacco user (6.3%) than those who did not (3.3%); no difference was observed by grade level. The prevalence of perceiving that SHS exposure causes “a lot of harm” was significantly higher among girls (38.7%) than boys (34.7%), among Hispanics (39.5%), non-Hispanic Asians (46.0%), and non-Hispanic Native Hawaiians/Pacific Islanders (47.6%) than non-Hispanic whites (34.7%), and among those who used no tobacco products (38.4%) than those who used combustible tobacco only (28.5%) or both combustible and non-combustible tobacco (24.1%); no difference was observed by grade level or whether a tobacco user lived in the home.

**Table 1:** Percentage of U.S. middle and high school students who think breathing smoke from other people’s cigarettes or other tobacco products causes ‘no’, ‘little’, ‘some’, or ‘a lot of’ harm, by selected characteristics - National Youth Tobacco Survey, 2012

**Characteristic**	**Unweighted frequency**	**No harm**		**Little harm**		**Some harm**		**A lot of harm**	
	**n**	**%**	**95% CI**	**%**	**95% CI**	**%**	**95% CI**	**%**	**95% CI**
**Sex**									
Girl	11,757	3.3	2.9-3.7	17.7	16.7-18.9	40.3	38.8-41.8	38.7	37.0-40.5
Boy	11,581	6.1	5.5-6.8	22.1	20.9-23.2	37.1	35.9-38.4	34.7	33.2-36.3
**Race/Ethnicity**									
White, Non-Hispanic	12,691	3.6	3.2-4.2	20.8	19.7-21.9	40.9	39.3-42.4	34.7	33.1-36.4
Black, Non-Hispanic	3,112	6.0	5.2-7.0	21.8	20.0-23.7	33.5	31.1-35.9	38.7	36.1-41.3
Hispanic	5,247	6.3	5.4-7.4	17.6	16.2-19.2	36.6	35.0-38.2	39.5	37.3-41.8
Asian, Non-Hispanic	1,141	2.9	2.0-4.2	13.5	11.1-16.2	37.6	34.0-41.4	46.0	42.0-50.0
American Indian/Alaska Native, Non-Hispanic	319	10.9	6.5-17.6	16.9	13.0-21.7	38.6	32.6-45.0	33.7	28.3-39.5
Native Hawaiian/Pacific Islander, Non-Hispanic	165	2.0	0.7-5.8	17.3	10.0-28.1	33.1	26.5-40.3	47.6	38.0-57.4
**Grade Level**									
Middle School	10,943	4.4	3.9-5.1	21.9	20.5-23.4	37.5	35.7-39.4	36.2	33.9-38.6
6^th^	3,368	4.2	3.4-5.2	22.2	19.9-24.7	37.3	34.9-39.9	36.3	33.6-39.1
7^th^	3,830	4.3	3.5-5.2	22.6	20.4-25.0	35.8	33.7-38.0	37.2	34.6-39.9
8^th^	3,745	4.8	3.9-5.7	20.8	18.2-23.7	39.4	36.2-42.7	35.1	30.7-39.6
High School	12,337	4.8	4.2-5.5	18.4	17.3-19.6	39.6	38.2-41.0	37.2	35.6-38.8
9^th^	3,110	6.1	5.0-7.4	19.8	18.0-21.8	39.1	36.8-41.4	35.0	32.5-37.7
10^th^	2,966	4.7	3.8-5.8	18.7	17.2-20.3	40.0	37.7-42.3	36.6	34.2-39.1
11^th^	3,210	4.2	3.4-5.1	17.7	16.1-19.5	39.8	37.7-42.0	38.3	35.8-40.8
12^th^	3,051	4.2	3.2-5.5	17.2	15.3-19.3	39.4	37.0-41.9	39.2	36.6-41.9
**Tobacco Use (Past 30 Days) **^**a**^									
No Tobacco Use	19,173	3.3	2.9-3.7	18.8	17.8-19.9	39.5	38.1-40.8	38.4	36.8-40.1
Non-Combustible Tobacco Use Only	2,491	4.7	2.7-7.9	23.4	19.2-28.3	39.7	33.3-46.4	32.2	25.9-39.3
Combustible Tobacco Use Only	311	9.1	7.7-10.8	25.1	23.2-27.1	37.3	35.2-39.4	28.5	26.5-30.5
Combustible and Non-Combustible Tobacco Use	869	19.6	15.8-23.9	28.3	24.2-32.8	28.0	24.2-32.1	24.1	21.0-27.6
**Lives with a Tobacco User **^**b**^									
No	13,096	3.3	2.8-3.8	18.5	17.6-19.4	40.4	38.9-41.8	37.9	36.3-39.6
Yes	9,788	6.3	5.6-7.2	21.5	20.2-22.9	37.1	35.6-38.6	35.0	33.3-36.7
**Overall**	23,346	4.7	4.3-5.2	19.9	19.0-20.9	38.7	37.5-39.9	36.7	35.3-38.2

*Abbreviation*: *CI* confidence interval.

Note: Row percentages may not add up to 100% due to rounding.

^a^ A respondent was considered to be a combustible tobacco user if they reported using cigarettes, cigars/cigarillos/little cigars, pipes, roll-your-own-cigarettes, bidis, clove cigarettes, hookahs or water pipes on at least 1 day during the past 30 days. Non-combustible tobacco users were defined as respondents who reported using chewing tobacco/snuff/dip, snus, or dissolvable tobacco products on at least 1 day during the past 30 days.

^b^ A respondent was considered to live with a tobacco user if they reported that anyone who lives with them now smokes cigarettes, uses smokeless tobacco such as chewing tobacco, smokes cigars, cigarillos, or little cigars, or uses any other form of tobacco.

The adjusted odds of perceiving that breathing SHS causes “some” or “a lot of” harm was significantly higher among non-Hispanic Asians (odds ratio [OR]=1.43, 95% CI=1.15-1.78), and among students in 10^th^ (OR=1.36, 95% CI=1.14-1.61), 11^th^ (OR=1.49, 95% CI=1.23-1.82), and 12^th^ (OR=1.69, 95% CI=1.38-2.08) grades (Table 2). The odds were lower among boys (OR=0.72, 95% CI=0.66-0.77), among non-Hispanic blacks (OR=0.83, 95% CI=0.74-0.93), among students who lived with a tobacco user (OR=0.81, 95% CI=0.75-0.87), and among those who used combustible tobacco only (OR=0.51, 95% CI=0.45-0.57) or both combustible and non-combustible tobacco products (OR=0.31, 95% CI=0.26-0.36).

**Table 2:** Percentage and adjusted odds ratios of U.S. middle and high school students who think breathing smoke from other people’s cigarettes or other tobacco products causes ‘some’ or ‘a lot’ of harm, by selected characteristics - National Youth Tobacco Survey, 2012

**Characteristic**	**Unweighted frequency**	**Percentage**		**Adjusted odds ratio **^**a**^	
	**n**	**%**	**95% CI**	**Odds ratio**	**95% CI**
**Sex**					
Girl	11,757	79.0	77.8-80.1	1.00	
Boy	11,581	71.8	70.5-73.1	**0.72**	**0.66-0.77**
**Race/Ethnicity**					
White, Non-Hispanic	12,691	75.6	74.3-76.8	1.00	
Black, Non-Hispanic	3,112	72.2	70.3-74.0	**0.83**	**0.74-0.93**
Hispanic	5,247	76.0	74.3-77.7	1.07	0.96-1.19
Asian, Non-Hispanic	1,141	83.6	80.7-86.2	**1.43**	**1.15-1.78**
American Indian/Alaska Native, Non-Hispanic	319	72.3	65.8-77.9	0.98	0.72-1.34
Native Hawaiian/Pacific Islander, Non-Hispanic	165	80.7	69.1-88.7	1.31	0.60-2.85
**Grade Level**					
Middle School	10,943	73.7	72.2-75.2		
6^th^	3,368	73.6	71.0-76.1	1.00	
7^th^	3,830	73.1	70.6-75.4	0.99	0.80-1.22
8^th^	3,745	74.4	71.4-77.3	1.08	0.86-1.35
High School	12,337	76.7	75.4-78.0		
9^th^	3,110	74.1	71.7-76.4	1.14	0.92-1.40
10^th^	2,966	76.6	74.7-78.4	**1.36**	**1.14-1.61**
11^th^	3,210	78.1	76.2-79.9	**1.49**	**1.23-1.82**
12^th^	3,051	78.6	76.7-80.4	**1.69**	**1.38-2.08**
**Tobacco Use (Past 30 Days) **^**b**^					
No Tobacco Use	19,173	77.9	76.8-78.9	1.00	
Non-Combustible Tobacco Use Only	2,491	71.9	66.6-76.6	0.79	0.62-1.02
Combustible Tobacco Use Only	311	65.8	63.5-68.0	**0.51**	**0.45-0.57**
Combustible and Non-Combustible Tobacco Use	869	52.1	48.1-56.1	**0.31**	**0.26-0.36**
**Lives with a Tobacco User **^**c**^					
No	13,096	78.3	77.3-79.3	1.00	
Yes	9,788	72.1	70.7-73.5	**0.81**	**0.75-0.87**
**Overall**	23,346	75.4	74.4-76.3		

*Abbreviation*: *CI* confidence interval.

^a^ Odds ratios were computed using a binary logistic regression model adjusted for all covariates listed in the table. Statistically significant odds ratios are noted in bold.

^b^ A respondent was considered to be a combustible tobacco user if they reported using cigarettes, cigars/cigarillos/little cigars, pipes, roll-your-own-cigarettes, bidis, clove cigarettes, hookahs or water pipes on at least 1 day during the past 30 days. Non-combustible tobacco users were defined as respondents who reported using chewing tobacco/snuff/dip, snus, or dissolvable tobacco products on at least 1 day during the past 30 days.

^c^ A respondent was considered to live with a tobacco user if they reported that anyone who lives with them now smokes cigarettes, uses smokeless tobacco such as chewing tobacco, smokes cigars, cigarillos, or little cigars, or uses any other form of tobacco.

### Discussion

This study is the first to assess the prevalence and predictors of SHS harm perceptions among a nationally representative sample of U.S. youth. The findings reveal that approximately three-quarters of U.S. middle and high school students perceive that SHS causes “some” or “a lot of” harm, but disparities in perceptions exist across subpopulations. Following multivariate adjustment, the perception that SHS causes harm was lower among boys, non-Hispanic blacks, those living with a tobacco user, exclusive combustible tobacco users, and concurrent users of combustible and non-combustible tobacco products.

These findings underscore the need for efforts to enhance knowledge of the hazards of SHS exposure among U.S. youth. Such efforts could include the expansion of smoke-free environments and public education campaigns on the hazards of SHS. Considerable progress has been made in increasing the number of U.S. states with comprehensive smoke-free policies that prohibit tobacco smoking in all indoor areas of worksites and public places, including restaurants and bars [8]. As of June 2013, 26 States and the District of Columbia had implemented comprehensive smoke-free policies and almost half (49.0%) of U.S. residents were covered by such policies at the state or local level [9,10]. Implementing comprehensive smoke-free policies is associated with more favorable attitudes toward the elimination of SHS exposure among adults, including smokers, and enhanced adoption of voluntary smoke-free home rules [11,12]. Similarly, youth living in communities with smoke-free laws and in households with voluntary smoke-free rules perceive smoking as less socially acceptable and are less likely to become established smokers [12]. In addition to the creation of smoke-free environments, education campaigns focused toward both adults and youth can help inform the public about the adverse health effects of SHS exposure [1]. For example, in 2012, the Centers for Disease Control aired “Tips from Former Smokers”, a nationwide paid-media campaign that featured real people suffering as a result of smoking and SHS exposure [13].

Strengths of this study include a large, nationally representative sample of middle and high school students and the ability to assess variations in perceptions across multiple subpopulations. However, the study is subject to at least two limitations. First, data were collected from youth enrolled in traditional middle or high schools and may not be representative of all youth. However, 98.5% of U.S. youth aged 10–13 years and 97.1% of those 14–17 years were enrolled in a traditional school in 2011 [14]. Second, data were self-reported and subject to potential recall or response bias.

### Conclusion

In conclusion, this study reveals that most U.S. middle and high school students perceive SHS as harmful, but efforts are needed to increase this knowledge among certain subpopulations, particularly tobacco users and those living with a tobacco user. Efforts to enhance knowledge of the hazards of SHS exposure could include expanding smoke-free environments and public education campaigns on the adverse health effects of SHS, which in turn may promote anti-smoking norms, reduce smoking, and improve health among U.S. youth.

## Abbreviations

CI: Confidence interval; NYTS: National Youth Tobacco Survey; OR: Odds ratio; SHS: Secondhand smoke.

## Competing interests

The authors have no conflicts of interest or financial disclosures to report. There were no sources of funding, either direct or indirect, for this study.

## Authors’ contributions

BK conceived of the study, conducted the analysis, and wrote the manuscript. SD provided input on the analysis, assisted with writing the manuscript, and reviewed the manuscript prior to submission. SB assisted with writing the manuscript and reviewed the manuscript prior to submission. All authors read and approved the final manuscript.
